# Biological barriers underlying the extremely high fatality of symptomatic rabies: neuroinvasion, immune evasion, and blood–brain barrier restriction

**DOI:** 10.3389/fimmu.2026.1904824

**Published:** 2026-09-04

**Authors:** Chen Sun, Xiao Guo, Liang Xiao, Wenxin Wang, Ge Shi, Chenci Liu, Shunyao Chen, Zhao-Hui Tang

**Affiliations:** 1 Department of Trauma Surgery, Wuhan Jinyintan Hospital, Tongji Medical College, Huazhong University of Science and Technology, Hubei Provincial Hospital for Infectious Diseases, Wuhan, China; 2 Animal-Related Injury Treatment Center, Wuhan Jinyintan Hospital, Tongji Medical College and State Key Laboratory for Diagnosis and Treatment of Severe Zoonotic Infectious Diseases, Huazhong University of Science and Technology, Wuhan, China; 3 Department of Trauma Surgery, Emergency Surgery & Surgical Critical, Tongji Trauma Center, Tongji Hospital, Tongji Medical College, Huazhong University of Science and Technology, Wuhan, China

**Keywords:** blood–brain barrier, fatal encephalopathy, immune evasion, neuroinvasion, post-symptom therapy, rabies virus, symptomatic rabies

## Abstract

Rabies remains one of the most preventable fatal infections, yet once clinical symptoms appear, survival is exceedingly rare. This paradox cannot be explained simply by the absence of a single effective antiviral drug. In this review, we frame symptomatic rabies as a sequential barrier-driven disease process in which neuroinvasion, immune evasion, and blood–brain barrier restriction converge to make post-symptom rescue biologically difficult. Rabies virus first changes the anatomical battlefield by entering peripheral nerves, exploiting receptor-supported uptake and retrograde axonal transport, and establishing infection within protected neural circuits. It then delays the host counterattack through weak or strain-dependent innate sensing, viral interference with interferon and signal transducer and activator of transcription (STAT) signaling, and insufficient conversion of peripheral immune activation into effective central nervous system clearance. Once central nervous system (CNS) infection is established, blood–brain barrier restriction further limits access of neutralizing antibodies, antiviral compounds, biologics, and immune cells to infected neural tissue. The convergence of these barriers permits CNS persistence and drives fatal encephalopathy, characterized less by widespread neuronal destruction than by synaptic, dendritic, circuit, autonomic, and functional collapse. Recent experimental advances, including CNS-accessible antibody strategies, blood–brain barrier (BBB)-modulating approaches, monoclonal antibody therapy, and One Medicine models, suggest that symptomatic rabies may require stage-specific therapeutic combinations rather than a single rescue intervention. We propose that future rabies therapy should be organized around three translational windows: preventing neuroinvasion before CNS entry, achieving immune-assisted viral control during early CNS infection, and combining CNS viral clearance with neuroprotective and autonomic support once encephalopathy is established. However, most mechanistic and therapeutic evidence remains derived from cellular and animal models, and clinical validation of CNS-directed or barrier-targeted interventions in symptomatic human rabies remains limited. Breaking the barrier cascade may be the central requirement for making symptomatic rabies treatable.

## 1 Introduction

Rabies is an acute, progressive encephalomyelitis caused predominantly by rabies virus (RABV), a highly neurotropic virus belonging to the genus *Lyssavirus*, subfamily *Alpharhabdovirinae*, family *Rhabdoviridae*, and order *Mononegavirales (*1). RABV is an enveloped, characteristically bullet-shaped virus containing an internal helical nucleocapsid surrounded by a host-derived lipid membrane. Its approximately 12-kb non-segmented, negative-sense single-stranded RNA genome encodes five major proteins in the conserved order 3′-N-P-M-G-L-5′: nucleoprotein (N), phosphoprotein (P), matrix protein (M), glycoprotein (G), and the large RNA-dependent RNA polymerase (L) (1). These structural and genomic features are closely linked to rabies pathogenesis: the surface glycoprotein mediates host-cell attachment and membrane entry, whereas several internal viral proteins contribute not only to replication and assembly but also to modulation of host antiviral responses.

Despite being preventable before neurological disease develops, rabies continues to cause an estimated 59,000 human deaths annually, mostly in Asia and Africa and often among communities with the least reliable access to post-exposure prophylaxis (PEP) (2, 3). The tools to stop most deaths are not imaginary: wound washing, timely vaccination, passive immunization for severe exposures, mass dog vaccination, surveillance, and One Health coordination have all proved their value (4, 5). Before the virus reaches the nervous system, rabies can still be prevented. After symptom onset, however, the therapeutic situation changes substantially. Once central nervous system (CNS) involvement becomes clinically apparent, human rabies remains nearly uniformly fatal, and there is still no WHO-approved diagnostic test that can reliably identify infection before symptom onset (4, 5). Ante-mortem diagnosis generally relies on a combination of viral RNA or antigen detection and rabies-specific antibody testing, with diagnostic performance influenced by disease stage and specimen type (6, 7). In this review, “symptomatic rabies” refers to rabies after the onset of clinically apparent neurological manifestations attributable to CNS infection, encompassing both furious and paralytic forms of the disease (8).

Immunological dysregulation is central to the progression of symptomatic rabies. Pathogenic RABV can limit early innate recognition and interferon responses, while viral proteins—particularly phosphoprotein P—interfere with TBK1- and STAT-dependent antiviral signaling (9–12). Compared with attenuated strains, pathogenic RABV generally induces weaker expression of interferon-stimulated genes, chemokines, and inflammatory mediators in the CNS (13). Consequently, peripheral immune activation may fail to translate into timely and effective clearance of infected neural tissue. This delayed and compartmentalized antiviral response contributes to viral persistence after neuroinvasion.

Recent reviews have clarified several parts of this problem, including the failure of post-symptom treatment protocols, the limits of induced coma and antiviral strategies, the role of immune evasion, and the difficulty of drug delivery into the CNS (9, 14–16). However, these mechanisms are often considered independently rather than as interacting stages of the same therapeutic problem, and how neuroinvasion, immune evasion, and BBB restriction interact sequentially to constrain therapeutic rescue remains insufficiently integrated. To address this gap, this review synthesizes current mechanistic and translational evidence within a unified three-barrier framework comprising neuroinvasion, immune evasion, and BBB restriction. These barriers respectively relocate infection into protected neural circuits, delay effective antiviral clearance, and restrict therapeutic access to infected CNS tissue. By linking their convergence to viral persistence, neuronal dysfunction, and stage-specific therapeutic opportunities, this framework aims to provide a more integrated explanation for the difficulty of treating symptomatic rabies after neural invasion.

The first barrier is neuroinvasion. Rabies virus does not merely infect neurons; it uses the nervous system as a protected route of spread. After inoculation, it can enter peripheral nerves, move by retrograde axonal transport, and establish infection within the central nervous system(CNS) (8). Recent receptor studies have refined this view: metabotropic glutamate receptor 2 (mGluR2) has been identified as a cellular receptor for rabies virus, and transferrin receptor 1 has been reported as an entry factor, suggesting that neural entry depends on a network of host factors rather than a single universal receptor (17, 18). Once this occurs, the battlefield changes. The target is no longer a contaminated wound, but an infection embedded in neural circuits.

The second barrier is immune evasion. Pathogenic rabies virus can cause devastating neurological disease without provoking an early, well-positioned immune response capable of clearing infected nervous tissue (9). Viral proteins, especially phosphoprotein P, interfere with interferon induction and downstream antiviral signaling; recent work shows that P protein can bind TANK-binding kinase 1 (TBK1) and disrupt innate immunity-related liquid condensates (10). The host response is not absent. It is late, misplaced, or functionally inadequate.

The third barrier is blood–brain barrier (BBB) restriction. Before neuroinvasion, peripheral neutralization can prevent disease. After CNS infection is established, potency alone is not enough. Experimental work suggests that BBB permeability and immune access are tightly linked to rabies outcome, and attenuated viruses may open the BBB more readily than wild-type pathogenic strains (19). Recent SynB1-conjugated antibody-cocktail data further underscore the same point: improving antibody penetration across the BBB can change therapeutic efficacy in lethal post-symptom models (16).

**Figure 1:**
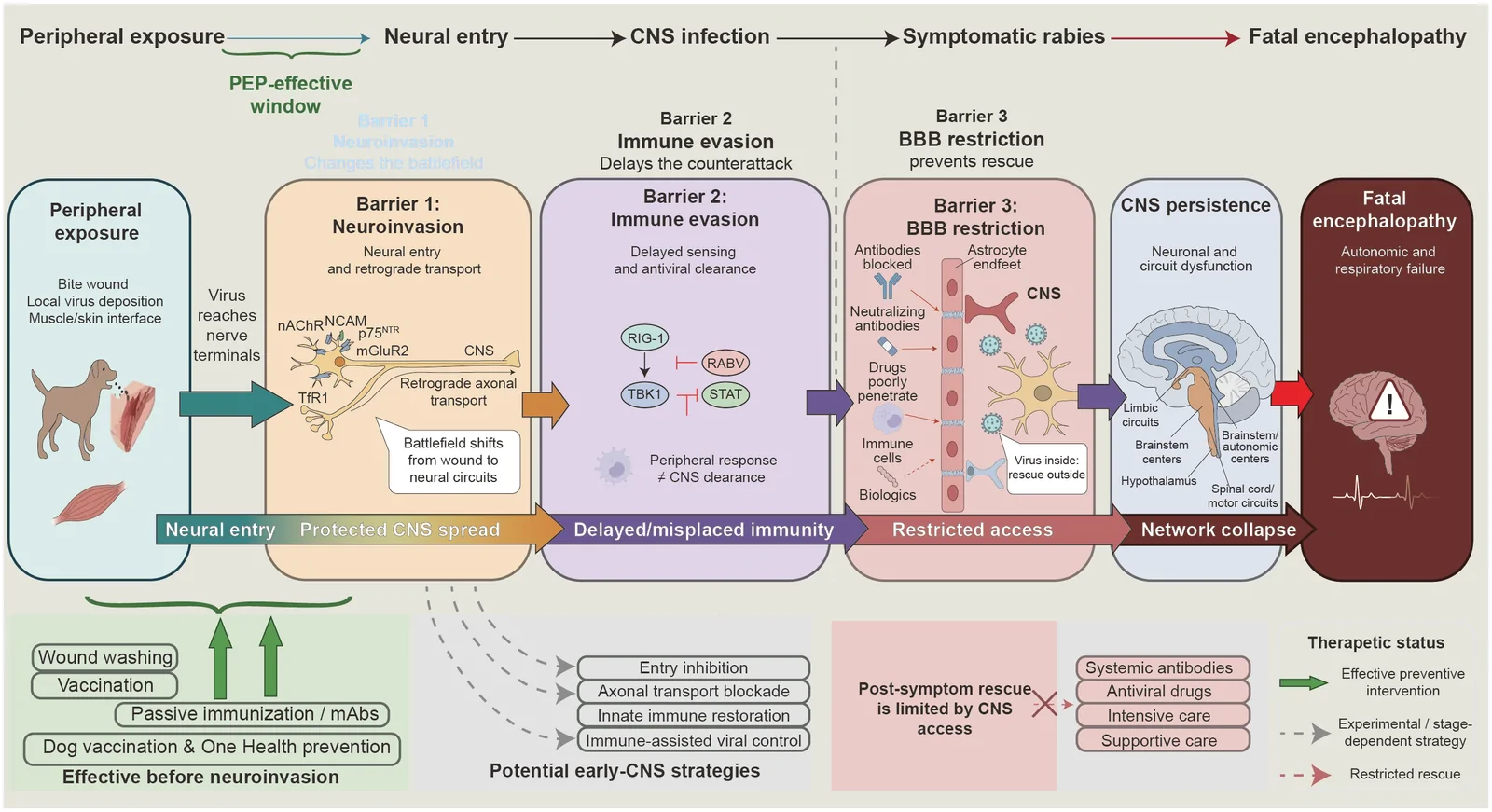
The three-barrier cascade underlying the extremely high fatality of symptomatic rabies. Rabies progresses from an accessible peripheral exposure to infection within protected neural compartments. Neuroinvasion shifts the infection into peripheral nerves and the central nervous system (CNS), immune evasion delays effective antiviral clearance, and blood–brain barrier (BBB) restriction limits the access of circulating antibodies, antiviral agents, biologics, and immune cells to infected CNS tissue. Together, these barriers permit viral persistence and progressive neuronal, autonomic, and respiratory dysfunction. Post-exposure prophylaxis remains highly effective before CNS entry, whereas treatment after symptom onset requires strategies capable of reaching and clearing infection within the CNS.

These barriers should not be read as isolated mechanisms. They form a chain: neuroinvasion changes the anatomical battlefield, immune evasion delays the counterattack, and BBB restriction prevents rescue from reaching the site of infection. This barrier cascade, and the shifting therapeutic windows along it, are summarized in **Figure 1**. Their convergence helps explain how a preventable peripheral exposure becomes fatal encephalopathy. If symptomatic rabies is ever to become treatable, the field will need to do more than manage the consequences. It will need to break the barriers.

## 2 Methods

This review was conducted as a structured narrative review. PubMed, Web of Science, and Google Scholar were searched for peer-reviewed studies relevant to rabies neuropathogenesis, neuroinvasion, immune evasion, blood–brain barrier regulation, neuronal dysfunction, diagnosis, and therapeutic intervention. Search terms included combinations of “rabies virus” or “RABV” with “neuroinvasion,” “neuronal entry,” “axonal transport,” “immune evasion,” “interferon,” “T cell,” “blood–brain barrier,” “CNS delivery,” “neuronal dysfunction,” “diagnosis,” and “therapy.” The search included publications available up to April 2026, without a lower date restriction in order to retain landmark mechanistic studies. Peer-reviewed original studies and relevant reviews in English were considered, with priority given to mechanistic studies, human data, and recent translational evidence. Conference abstracts, preprints, and articles without direct relevance to the biological or therapeutic mechanisms discussed in this review were excluded.

## 3 Neuroinvasion changes the battlefield

Rabies begins as an exposure at the skin or muscle, but it becomes a different disease once the virus enters the nervous system. At the wound, intervention still has room to work. The length of that window is uneven: incubation varies with inoculum, wound severity, anatomical site, innervation density, and distance from the central nervous system (CNS); highly innervated or cranial exposures leave less time for error (8, 20). The incubation period is therefore not a harmless silent phase. It is the interval in which prevention can still beat neuroinvasion.

Once RABV enters peripheral nerves, the therapeutic target changes. A contaminated wound is accessible. An infected axon is not. Human disease already hints at this shift. In dog-variant rabies summarized from Thai cohorts, furious rabies accounted for roughly two-thirds of cases and paralytic rabies for about one-third; without full intensive-care support, mean survival from clinical onset to death was 5.7 days in 80 furious cases and 11 days in 35 paralytic cases (8). More unsettling, RABV may already be present in the CNS before the classical encephalitic syndrome becomes obvious: subclinical anterior horn cell dysfunction and abnormal brain MRI findings can precede brain symptoms in furious rabies (8). Clinical manifestations may therefore lag behind the establishment of CNS infection. By the time the full syndrome appears, the battlefield has usually moved.

The entry step has never fit neatly into a single-receptor model. Early studies implicated the nicotinic acetylcholine receptor at the neuromuscular junction, neural cell adhesion molecule, and p75 neurotrophin receptor as candidate receptors or attachment factors (21–23). These discoveries were important, but none explains the full range of RABV tropism across tissues, strains, and neuronal populations (24–26). p75 neurotrophin receptor (p75NTR) is a useful cautionary example: it can bind RABV glycoprotein and support receptor-linked axonal trafficking, yet it is not absolutely required for infection (24, 27). More recent work supports a receptor-network view. mGluR2 has been identified as a cellular receptor for RABV (17), and transferrin receptor 1 (TfR1) has been reported as a functional entry factor (18). TfR1 knockdown strongly inhibited ERA- enhanced green fluorescent protein (EGFP) infection in HEK293 and N2a cells, while anti-TfR1 antibody or soluble TfR1 ectodomain protein reduced infection in HEK293 cells, N2a cells, and mouse primary neurons (18). TfR1 also interacts with RABV G, is internalized with the virus, and accompanies RABV into Rab5- and Rab7-positive endosomal compartments (18).

Receptor engagement is followed by an active endocytic process rather than passive viral uptake. Experimental studies indicate that clathrin-mediated endocytosis represents a major route of rabies virus (RABV) internalization. In epithelial cells, viral particles are internalized through elongated, incompletely coated clathrin pits, and actin polymerization is required to complete membrane envelopment and productive uptake (28). Live-cell imaging in Vero cells similarly demonstrated endocytic uptake of RABV particles followed by trafficking through acidic intracellular compartments (29). In N2a neuronal cells, CVS-11 entry was shown to depend on clathrin, membrane cholesterol, dynamin, and a low-pH environment, while caveolae were not required (30). Studies in primary dorsal root ganglion and ventral spinal cord neurons further showed that RABV glycoprotein-mediated uptake is predominantly clathrin- and dynamin-dependent (31). Pharmacological inhibition of macropinocytosis reduced infection in some neuronal preparations, although the effect was weaker and less consistent than inhibition of dynamin-dependent uptake, and limited colocalization with fluid-phase endocytic markers suggested that macropinocytosis is unlikely to represent the dominant neuronal entry pathway (31). Thus, macropinocytic uptake may contribute in a cell-type-dependent or auxiliary manner rather than serving as a universal mechanism of RABV entry.

Membrane fusion constitutes a subsequent and spatially regulated step in productive neuronal infection. After internalization at distal neuronal processes, incoming virions can remain within endosomal carriers during retrograde transport, whereas efficient release of the viral ribonucleoprotein complex requires endosomal acidification and RABV glycoprotein-mediated membrane fusion near the neuronal cell body (31). Cytoskeletal dynamics therefore participate at multiple stages of neuroinvasion. Actin supports completion of endocytic internalization (28), whereas microtubule-dependent trafficking subsequently transports virus-containing endosomes toward the neuronal soma. More recent evidence further shows that RABV infection induces dynamic remodeling of the actin cytoskeleton through Rac1-dependent signaling involving the Pak1–Limk1–Cofilin1 axis, with perturbation of this pathway exerting particularly strong effects during the viral entry phase (32). These findings indicate that the cytoskeletal and endocytic state of a target cell may influence RABV entry efficiency and could therefore constitute a cell-dependent host restriction point during early infection.

After entry, neurons provide RABV with something most tissues cannot: a long-distance transport system. In compartmentalized dorsal root ganglion cultures, EGFP-labeled RABV added only to distal axons moved retrogradely toward neuronal cell bodies. Among 244 tracked particles, 87.29% met criteria for directed movement (27). The virus also moved faster than a physiological neurotrophin cargo: average RABV speed was about 40% higher than nerve growth factor (NGF) speed, 0.93 ± 0.03 μm/s versus 0.66 ± 0.02 μm/s (27). This is not simple hitchhiking. The virus appears to make the ride more efficient.

The axonal route is also resistant to some local antiviral cues. In tri-chamber superior cervical ganglion cultures, IFNβ and IFNγ were biologically active when applied to neuronal cell bodies, reducing infection from 47.0 ± 5.3% to 33.4 ± 10.9% and 8.83 ± 6.8%, respectively (33). Yet local axonal IFN treatment did not meaningfully alter retrograde transport: untreated axons carried 5.4 ± 4.7 moving particles per field of view, compared with 4.7 ± 3.4 after IFNβ and 5.0 ± 4.2 after IFNγ (33). Emetine behaved differently, reducing moving RABV particles from 5.0 ± 3.9 to 1.5 ± 0.9 per field of view while largely preserving Rab5- and Rab7-positive vesicle transport (33). The route is not untouchable. But it is specialized.

Retrograde movement remains central, but RABV transport is not perfectly one-directional. In compartmentalized dorsal root ganglion neurons, entry-related retrograde transport occurred at about 1.5 μm/s. After two days of infection, newly generated particles moved in both directions: postreplicative retrograde transport was similar, around 1.6 μm/s, whereas anterograde transport was faster, about 3.4 μm/s (26). Particles lacking glycoprotein did not move anterogradely, indicating that this later movement depends on RABV G (26). This does not weaken the retrograde model. It shows that once neuronal infection is established, the virus can exploit neuronal polarity in more than one direction.

Newer neuron-focused studies sharpen what “neural battlefield” means, but the key point is not neuron biology for its own sake. In a single-nucleus RNA-seq study of mouse visual cortex, 8,745 nuclei obtained from RABV-infected cortical samples and 9,508 uninfected control nuclei were analyzed. Nuclei from infected samples could still be assigned to cortical cell classes and subclasses, yet RABV infection was associated with marked transcriptional changes, with up-regulated genes enriched for viral response, type I interferon signaling, cytokine response, and NF-κB pathways, and down-regulated genes involving respiration, metabolism, synaptic compartments, and somatodendritic compartments (24). Human induced pluripotent stem cell (hiPSC)-derived neural cultures point in the same direction: RABV-TH infected neural progenitor cells, neurons, and astrocytes, but induced apoptosis in neural progenitor cells rather than in infected neurons or astrocytes under the same conditions (34). Proteomic profiling of infected hiPSC-derived neurons further showed time-dependent changes in neurotransmitter transport, ion transport, trans-synaptic transmission, cytoskeletal regulation, and synaptic signaling (34). Neuroinvasion is therefore not simply occupation of an anatomical space. It is occupation of a living, signaling network that the virus can perturb without immediately destroying.

Neuroinvasion is the first biological barrier to rescue. It converts an accessible peripheral exposure into a distributed infection moving through receptors, endosomes, axons, synapses, and altered neural circuits. Before this transition, prevention can act at the wound, in the circulation, and in lymphoid tissues. After it, antibodies and drugs face a moving target inside protected cellular architecture. The virus has not just moved inward. It has changed the rules of engagement.

That shift sets up the next problem. Once RABV is inside the nervous system, survival depends on whether the host can recognize and clear infection in an anatomically secluded and immunologically unusual compartment. In pathogenic rabies, that counterattack is often late, weak, or poorly positioned. Neuroinvasion changes the battlefield; immune evasion determines how long the virus can hold it.

## 4 Immune evasion delays the counterattack

If neuroinvasion changes the battlefield, immune evasion determines whether the host can respond before the virus settles in. In symptomatic rabies, that response is usually too late or too poorly placed to matter. This does not mean that the immune system is absent. It means something more frustrating: the signal is weak when it should be loud, and by the time it becomes detectable, the virus has often moved into a compartment where clearance is much harder. Pathogenic RABV can advance through peripheral nerves and into the CNS while provoking only limited early inflammation, in marked contrast to attenuated strains that more readily activate innate immune programs (9, 13, 35).

The difference between pathogenic and attenuated strains is not just a convenient laboratory contrast. It has become one of the clearest windows into rabies immune evasion. In the CNS, attenuated RABV induces stronger expression of interferon-related genes, chemokines, and inflammatory mediators, whereas pathogenic strains show a much quieter profile (13, 36). A more recent comparison of the pathogenic Tha strain and the vaccine SAD strain makes the point at the RNA-sensing level. RABV RNAs were detected mainly through retinoic acid-inducible gene I (RIG-I), but the immunological output differed sharply by strain: compared with Tha infection, SAD infection induced significant upregulation of 16 interferon-stimulated genes, including IFI16, IFI35, IFI44L, IFITM2, IFITM3, IRF7, ISG15, OAS2, STAT1, STAT2, TAP1, PML, and UBE2L6 (37). The same study identified two 5′ copy-back defective interfering genomes of 2170 and 1668 nucleotides in SAD-infected cells, but not in Tha-infected cells; *in vitro*-transcribed DI-1668 and DI-2170 RNAs strongly activated an interferon-stimulated response element reporter (37). So the problem is not merely that wild-type virus blocks immunity. In some settings, it is poorly seen in the first place.

At the protein level, phosphoprotein P remains the best-characterized immune-evasion factor. Classical studies showed that P interacts with STAT1 and STAT2 and blocks IFN-α/β- and IFN-γ-triggered JAK–STAT signaling, in part by preventing activated STATs from reaching the nucleus (11, 12). Later work extended this to STAT3/gp130 signaling and to the nuclear trafficking determinants of P (38, 39). The newer TBK1 study adds a useful upstream mechanism. PSAD, but not PCVS, inhibited ΔRIG-I-driven IFN-β promoter activation; switching residue 179 was enough to change the phenotype, with S179P abolishing the inhibitory activity of PSAD and P179S restoring inhibition in PCVS (10). Mass spectrometry and co-immunoprecipitation then identified TBK1 as a preferential partner of inhibitory P forms, and the non-inhibitory SAD-S179P P protein showed about three-fold less TBK1 association (10). Even more interesting, TBK1, NAP1, and SINTBAD formed dynamic cytoplasmic condensates after innate immune stimulation, and P–TBK1 interaction interfered with their formation (10).

Other structural proteins contribute to this quieting, although less spectacularly. The nucleoprotein N can shield viral RNA from RIG-I-mediated recognition and reduce induction of IFN and chemokines (40, 41). The matrix protein M has its own route into innate signaling: field-isolate M proteins can target RelAp43, a RelA splice variant involved in NF-κB-dependent antiviral responses (42, 43). In Luco and colleagues’ study, RelAp43 transcription rose roughly 2- to 3.5-fold by 48 h after lyssavirus infection, but field-isolate M-Tha reduced NF-κB-dependent and IFN-β promoter activity more strongly than vaccine-strain M-SAD; RelAp43 knockdown reduced IFN-β mRNA induction by about half after Tha or SAD infection (43). Glycoprotein G is not just an entry and neutralization antigen either. Recombinant viruses carrying the wild-type G ectodomain showed poor dendritic-cell binding and activation compared with laboratory-adapted counterparts, indicating that the G ectodomain can help determine whether antigen-presenting cells are efficiently engaged (44, 45).

Innate immune signaling in the CNS is not uniformly protective and may also contribute to rabies-associated neuropathology. Toll-like receptor 3 (TLR3) participates in viral RNA sensing and has been implicated in rabies pathogenesis and Negri body formation. In a mouse model, pharmacological inhibition of TLR3 signaling altered inflammatory responses and pathological progression, supporting a functional role for this pathway during CNS infection (46). The MEK–ERK1/2–MAPK pathway also contributes to RABV-induced pathology; its inhibition reduced viral-associated pathological changes and modulated pro-inflammatory mediators in infected mice (47). In addition, RABV infection has been associated with increased inducible nitric oxide synthase, TNF-α, caspase-1, FasL, and TLR3 expression in the brain, whereas inhibition of inducible nitric oxide synthase delayed disease progression and reduced several inflammatory and apoptotic mediators (48). Together, these findings indicate that CNS antiviral signaling involves a balance between viral restriction and inflammatory injury rather than a simple deficiency of immune activation.

The peripheral innate response is just as important, and here the picture is more nuanced than simple suppression. Human monocyte-derived macrophages exposed to street RABV for 48 h did not merely become inert. Transcriptomic analysis showed a distinct polarization program: among 8986 substantially expressed genes, 5921 were shared across macrophage phenotypes, but 246 were specific to the RABV-exposed state; compared with M0 macrophages, RABV macrophages had 2773 differentially expressed genes, fewer than M1 macrophages but far more than M2a or M2c macrophages (49). The RABV profile included antiviral and interferon-related genes such as APOBEC3A, IFIT family members, OAS1–3, ISG15, MX1, MX2, IRF7, and multiple TRIM genes, yet also contained IL10 and other potentially regulatory elements (49).

The dendritic-cell data are even more revealing. In a 2025 human monocyte-derived dendritic cells study, SHBRV and SAD P5 infected only a small fraction of cells: less than 1% at multiplicity of infection (MOI) 0.1 and 4.8–10.9% at MOI 5, while dog-associated RABV produced no detectable dendritic cells infection (50). SHBRV and SAD P5 increased CD80, CD86, HLA-DR, and PD-L1 expression mainly at high MOI, but the overall response remained modest. Cytokines such as TNF-α, CXCL10, IL-6, IFN-λ1, IFN-β, and IFN-α2 rose significantly after SHBRV exposure at MOI 5, yet stayed well below LPS-induced levels (50). The functional endpoint was blunt: RABV-exposed dendritic cells did not enhance naïve T cell proliferation beyond mock-exposed controls, with proliferation around 5% compared with 12.5% after LPS stimulation (50). The cells noticed something. They did not launch a strong adaptive response.

**Figure 2:**
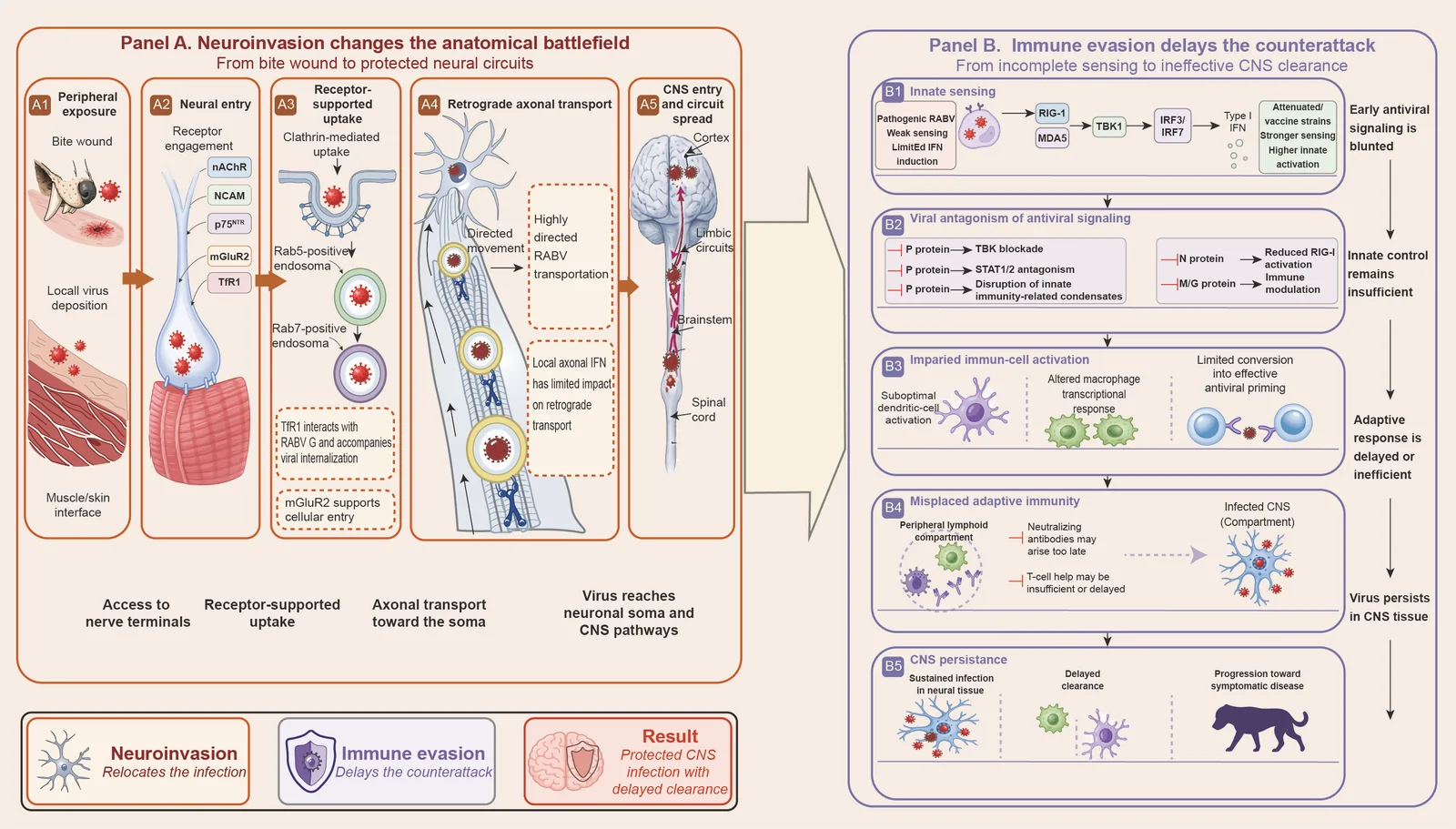
Neuroinvasion and immune evasion reshape the anatomical and immunological battlefield. **(A)** Summarizes the transition from peripheral exposure to neural infection. Rabies virus (RABV) engages multiple neuronal entry factors, undergoes predominantly clathrin- and dynamin-dependent endocytosis, and subsequently requires endosomal acidification and glycoprotein-mediated membrane fusion. Actin remodeling contributes to internalization, whereas microtubule-dependent trafficking supports retrograde transport toward the neuronal soma and CNS. Macropinocytosis may provide an auxiliary, cell-dependent entry route. **(B)** Summarizes the major mechanisms that delay effective antiviral clearance after neural invasion, including weak or delayed viral sensing, viral interference with interferon and related signaling pathways, and insufficient coordination of antigen-presenting cells and T-cell responses. Together, these mechanisms facilitate persistent infection within neural tissue.

That distinction matters for symptomatic rabies. Adaptive immunity is also important once infection reaches the CNS. CD4^+^ T cells can contribute to antibody-mediated clearance of CNS-resident lyssavirus (51), whereas effective CD8^+^ T-cell responses depend on appropriate antigen presentation and access to infected neural tissue. However, excessive or poorly regulated lymphocyte infiltration may also amplify CNS inflammation, emphasizing that successful immune control requires both antiviral efficacy and spatial regulation. Peripheral immunity does not automatically become CNS immunity, and a late serum neutralizing antibody response does not necessarily mean virus clearance from infected brain tissue (8, 36, 51). Immune evasion therefore acts as the second biological barrier to rescue. Neuroinvasion moves RABV into a protected anatomical space; immune evasion gives it time there. **Figure 2** summarizes how RABV moves the infection into protected neural circuits and then delays effective CNS immune clearance. By the time the counterattack forms, it still has to cross another boundary. That boundary is the blood–brain barrier.

## 5 Blood–brain barrier restriction prevents rescue

The blood–brain barrier is usually presented as a defender of the central nervous system. In symptomatic rabies, that description becomes uneasy. The same interface that protects neural tissue from circulating toxins, pathogens, and excessive inflammation can also keep therapeutic molecules and immune effectors away from infected brain tissue (52–54). This is not a minor pharmacological inconvenience. Once RABV has established infection in the CNS, the BBB becomes part of the disease problem. It turns rescue into a delivery crisis.

The barrier is not a wall, of course. It is a regulated neurovascular unit built from non-fenestrated brain endothelial cells, tight junctions, adherens junctions, pericytes, astrocytic endfeet, basement membrane, transporters, and efflux systems (52–54). Its selectivity is the point. More than 98% of small-molecule drugs and nearly all large-molecule therapeutics are estimated to fail to penetrate the brain sufficiently for pharmacological effect, and antibodies often reach only 0.01–0.1% of their circulating concentrations in brain tissue (54). For normal brain physiology, this is elegant protection. For symptomatic rabies, it is a trap. Neutralizing antibodies, antiviral compounds, biologics, and immune cells may exist in the blood but still fail to reach infected neural tissue in time or in sufficient amounts (8, 36, 55). Therapeutic potency alone is insufficient if adequate drug exposure cannot be achieved within the infected CNS compartment.

Rabies makes this problem unusually visible because survival appears to depend on whether immune effectors can enter the CNS. In silver-haired bat rabies virus infection, Hooper and colleagues showed that lethal outcome was linked to failure to open the BBB and deliver immune effectors into CNS tissues (55). A related study then showed that lethal infection could be prevented by opening the BBB, strengthening the idea that peripheral immunity becomes useful only when it can become brain immunity (56). Roy and Hooper later framed this as an immune-evasion strategy: pathogenic RABV tends to maintain BBB integrity, whereas attenuated viruses more readily permit immune access (36). The question is no longer just “does the host respond?” The better question is: can that response get in?

The contrast between wild-type and attenuated RABV has provided the strongest experimental handle. Laboratory-attenuated strains induce more chemokines and cytokines in the CNS, promote leukocyte infiltration, and increase BBB permeability, whereas wild-type strains generally provoke milder inflammation and preserve restriction (57–59). In Chai and colleagues’ study, BBB permeability was enhanced in mice infected with laboratory-attenuated RABV but not wild-type RABV, and this was accompanied by reduced expression of claudin-5, occludin, and ZO-1 in brain microvasculature (57). Importantly, RABV did not directly infect brain microvascular endothelial cells or directly alter their tight-junction proteins; instead, brain extracts from attenuated RABV-infected mice, rich in inflammatory chemokines and cytokines, reduced tight-junction expression in endothelial cells (57). That result changes the mechanism. The BBB opens not simply because virus touches the endothelium, but because infection reshapes the inflammatory environment around it.

C-X-C motif chemokine ligand 10 (CXCL10) gives this cascade a more anatomical shape. Neuronal CXCL10 expression was detected as early as 3 days post-infection, before its later detection in microglia at 6 days and astrocytes at 9 days (58). Neutralizing CXCL10 reduced IFN-γ production, Th17 infiltration, tight-junction loss, and BBB permeability (58). The matrix metalloproteinase 8 (MMP8) story adds another layer. Fang and colleagues found that MMP8 was upregulated in brains infected with laboratory-attenuated CVS-B2c but not dog-derived wild-type DRV-Mexico; MMP8 expression increased across brain regions including cortex, cerebellum, and brainstem, and was induced in BV2 microglia, C8 astrocytes, and N2a neuronal cells after CVS infection (19). *In vitro*, MMP8 protein reproduced tight-junction degradation, whereas an MMP8 inhibitor blocked this effect (19). These findings indicate that increased BBB permeability may facilitate immune access but is mediated through inflammatory and proteolytic pathways that can also contribute to tissue injury.

This is why “BBB restriction” is more accurate than “BBB failure” for symptomatic rabies. The problem is often not that the barrier collapses. It is that the barrier remains restrictive after the virus has already crossed into the CNS. Neutralizing antibody is the clearest example. It is indispensable for prevention when present early, but after CNS infection is established, serum virus-neutralizing antibody does not guarantee useful antibody exposure in brain parenchyma (36, 55, 60). Huang and colleagues directly tested this therapeutic logic: enhancement of BBB permeability was required for intravenously administered virus-neutralizing antibodies to clear established RABV infection from the brain and prevent rabies in mice (60).BBB modulation should therefore be considered a double-edged therapeutic strategy. Controlled and transient increases in permeability may facilitate the entry of neutralizing antibodies and antiviral immune effectors into infected CNS tissue (60), whereas excessive or prolonged barrier disruption may enhance leukocyte infiltration, inflammatory cytokine exposure, and secondary neural injury (57, 58). The therapeutic objective should therefore be selective and temporally controlled CNS access rather than indiscriminate BBB opening.

Local delivery tries to bypass the problem. Intrathecal or intraventricular administration is attractive because it moves treatment closer to the infected compartment. Yet it remains clinically awkward: uneven parenchymal distribution, uncertain timing, procedural risk, and late recognition of disease all limit its usefulness (14, 15). General BBB-delivery strategies—receptor-mediated transcytosis, transferrin receptor targeting, VNAR-based shuttles, peptide conjugation, and nanocarriers—have expanded the toolkit, but most remain far from standard rabies therapy (54, 61, 62). The vocabulary is changing, though. The field is moving from “stronger antibody” toward “brain-accessible antibody.”

The SynB1-conjugated antibody-cocktail study makes that shift concrete. In that model, RABV reached the brain by about 5 days post-infection, and treatment was given when CNS invasion and symptoms were evident (63). A SynB1-conjugated triple antibody cocktail targeting different RABV-G epitopes produced 80% survival in CVS- or DRV-infected mice when administered at 5 dpi, whereas the unconjugated cocktail achieved only 20% survival in CVS infection and 0% in DRV infection (63). Treated mice showed markedly lower viral genomic RNA levels in spinal cord, brainstem, cerebellum, and cerebrum, with minimal RABV-positive cells by immunostaining (63). Human BBB biology is more complex, and safety, pharmacokinetics, tissue distribution, and off-target delivery will need rigorous testing. But the principle is hard to miss: CNS delivery can change outcome.

**Figure 3:**
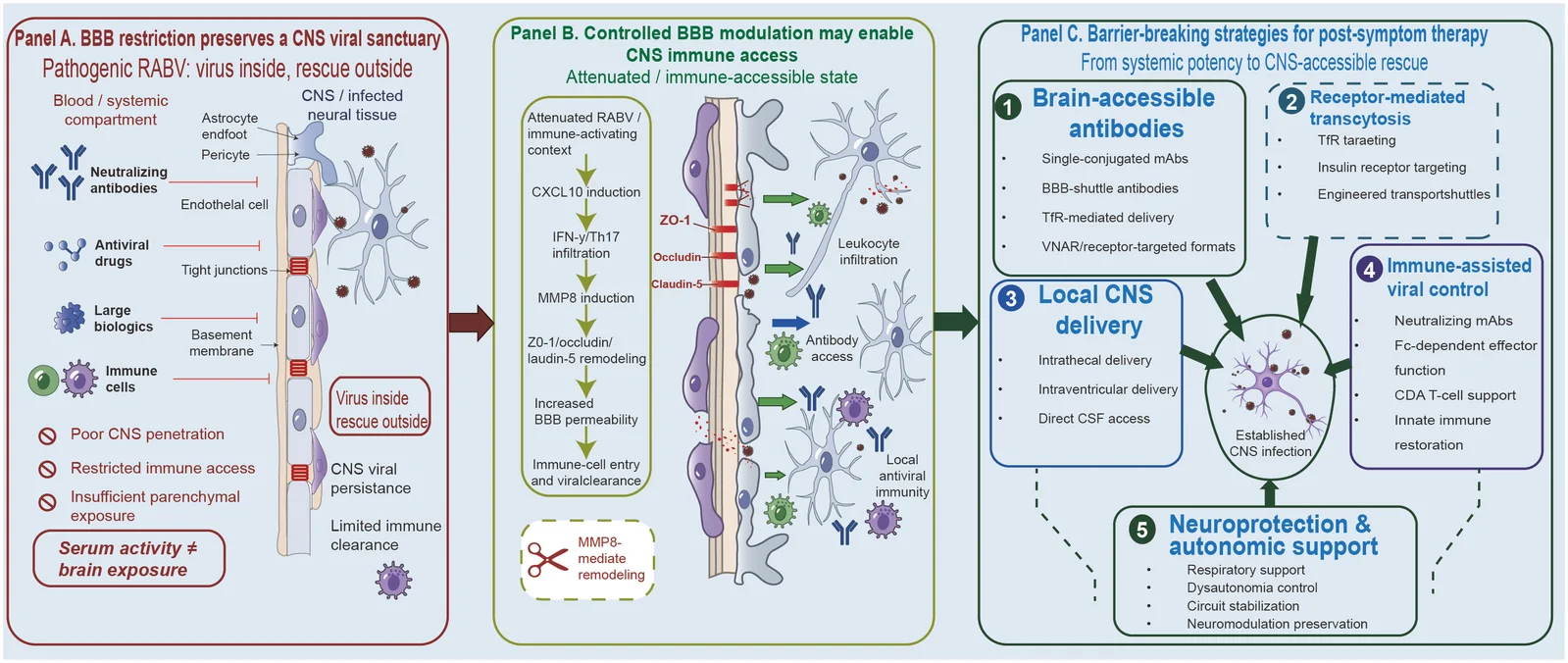
Blood–brain barrier restriction and therapeutic strategies to break the rescue barrier. **(A)** Illustrates the spatial mismatch that develops after RABV establishes infection within the CNS: circulating antibodies, antiviral agents, biologics, and immune cells may have limited access to infected neural tissue because of blood–brain barrier (BBB) restriction. **(B)** Illustrates the double-edged nature of BBB modulation. Controlled and transient increases in CNS access may facilitate delivery of antiviral antibodies and immune effectors, whereas excessive barrier disruption may promote inflammatory-cell infiltration and secondary neural injury. **(C)** Summarizes major therapeutic directions, including brain-accessible antiviral agents and antibodies, targeted or local CNS delivery strategies, and combined approaches integrating viral clearance with controlled immune responses and neuroprotective or autonomic support. Effective treatment is therefore likely to require sufficient CNS exposure without uncontrolled disruption of BBB homeostasis.

BBB restriction is therefore the third biological barrier to rescue. Neuroinvasion determines where the virus hides. Immune evasion explains why it persists. BBB restriction explains why rescue cannot easily reach it. **Figure 3** illustrates the core rescue problem in symptomatic rabies: the virus is inside the CNS, while most therapeutic tools remain outside. By the time symptoms declare themselves, the virus is already inside a protected neural compartment, the counterattack has been delayed or misplaced, and the main therapeutic tools remain largely outside the battlefield. This creates a spatial mismatch between CNS-resident infection and systemically delivered therapeutic effectors. This compartmental mismatch represents a major therapeutic obstacle in symptomatic rabies.

## 6 Barrier convergence: from CNS persistence to fatal encephalopathy

Neuroinvasion, immune evasion, and blood–brain barrier restriction are not three separate explanations for death in rabies. They are parts of the same failure. CNS entry alone would not be uniformly fatal if infected tissue could be rapidly recognized and cleared; immune evasion would be more tractable if antibodies and effector cells could freely enter the infected compartment; BBB restriction becomes decisive only after the virus has already shifted the disease into neural tissue. The barriers are encountered in sequence, but they behave as a system (8, 64, 65).

The downstream disease is therefore not just “viral encephalitis” in a generic sense. Whole-brain fMOST mapping has shown that RABV distribution is broad and route-dependent, with prominent infection in cortex, hypothalamus, hippocampus, midbrain, and hindbrain; intramuscular inoculation concentrated virus in motor cortex and the head of the striatum, whereas intranasal inoculation favored the dentate gyrus (66). Some circuit involvement was less route-dependent. Nuclei linked to fear processing, including the central amygdala, bed nucleus of the stria terminalis, periaqueductal gray, and basolateral amygdala, were extensively infected regardless of inoculation route (66). That matters clinically. Hydrophobia, aerophobia, agitation, dysautonomia, and respiratory failure are not random symptoms; they map onto disturbed limbic, brainstem, hypothalamic, and autonomic circuits (66, 67).

A long-standing paradox still holds: rabies can devastate brain function without producing proportional histological destruction. Human studies suggest that neuronal apoptosis is not a major pathogenetic feature in most rabies encephalitis (64). In a street-virus hippocampal model, MRV antigen appeared in CA1 pyramidal neuronal cell bodies and processes by day 4 post-infection, but by day 7 was mainly located in somata; hematoxylin and eosin staining and microtubule-associated protein 2 (MAP2) staining still showed no well-defined cytopathological damage to neuronal bodies or dendrites (68). The synaptic compartment told a different story. In primary hippocampal neurons, spine density fell from 12 ± 3.0 to 4 ± 2.0 in the authors’ quantification, and this loss was accompanied by F-actin depolymerization (68). The neuron did not need to disappear. Its connections were already failing.

Other models point to the same level of vulnerability. In the spinal cord, infected mice showed statistically significant changes in MAP2 and neurofilament heavy chain immunoreactivity, with optic densitometry differences reaching P = 0.0135 for MAP2 and P = 0.0331 for neurofilament heavy chain (69). Golgi–Cox staining added the more important morphological detail: ventral horn neurons from infected mice had fewer dendritic branches and shorter dendrites, even though the average soma diameter remained similar between control and infected animals, 23.81 µm versus 21.69 µm (69). This fits the clinical problem of paralytic rabies better than a simple “neurons die” model. Motor circuits can fail when dendrites, cytoskeleton, and axonal signaling are distorted.

Functional evidence is now catching up with morphology. In differentiated human neurons, early RABV infection modulated genes involved in neuronal function, signaling, immune response, and cell-cycle-related pathways; the overall DEG range was relatively modest, consistent with a stealthy but biologically meaningful perturbation (70). Calcium imaging then gave the transcriptomic signal physiological weight: Tha- and Th4M-infected neurons showed reduced spontaneous activity compared with noninfected neurons, and γ-aminobutyric acid (GABA) treatment further reduced calcium peak frequency in infected cultures (70). These are not terminally lysed cells. They are living neurons with altered excitability and network behavior.

The older “dysfunction without injury” view, however, should not be overstated. Some injury programs appear under specific experimental conditions. In primary neuronal cultures infected with CVS, more than 90% of neurons expressed viral antigen; TUNEL positivity appeared by 24 h post-infection, activated caspase-3 was detected, and the caspase inhibitor DEVD-CHO significantly improved viability (71). A newer study extended this injury framework to pyroptosis: RABV infection upregulated Gsdmd, Nlrp3, caspase-1, IL-1β, and several chemokines, and GSDMD protein rose to 691.1–5764.96 pg/mL in neurons infected with different RABV strains, compared with less than 100 pg/mL in mock-infected neurons (72). These data do not overturn the functional-collapse model. They refine it. Fatal rabies is mainly a disorder of neural circuit failure, but selective cell-death programs may contribute depending on strain, route, timing, and tissue context.

This helps explain why supportive care so often reaches a ceiling. Ventilation can support breathing; sedation can control agitation; hemodynamic management can temporarily stabilize dysautonomia. But none of these removes virus from infected circuits, restores effective CNS immunity, or solves BBB-restricted delivery (14, 15). The Milwaukee protocol’s failure is a clinical reminder of the same biology: induced coma and nonspecific neuroprotection do not break the barrier cascade (15). By the time advanced symptoms appear, neuroinvasion has defined the protected battlefield, immune evasion has permitted persistence, and BBB restriction has kept rescue at a distance. Fatal encephalopathy is the product of their convergence.

## 7 Breaking the barriers: future perspectives

The extremely high fatality of symptomatic rabies should make the field suspicious of simple rescue narratives. A stronger antiviral would be welcome, but potency alone is not the problem. By the time symptoms appear, RABV has usually entered neural tissue, delayed the host response, and became partly shielded from systemic therapy by the blood–brain barrier. Future treatment therefore needs to be framed less as a search for one decisive drug and more as a staged attempt to break the barrier cascade (14, 15, 73). As summarized in **Figure 3**, the central therapeutic challenge is not only to identify antiviral agents, but to deliver them into infected CNS compartments at the appropriate stage of disease.

Timely diagnosis is an additional translational constraint because successful barrier-targeted therapy depends on recognizing CNS infection before irreversible neurological dysfunction develops. Current ante-mortem diagnosis relies on combinations of viral RNA or antigen detection from saliva or nuchal skin specimens together with antibody testing in serum or cerebrospinal fluid, but diagnostic sensitivity varies with disease stage and specimen type (6, 7). Importantly, there remains no validated biomarker that reliably identifies the transition from peripheral infection to early CNS involvement before overt neurological symptoms. Improved molecular, immunological, or neuroinjury biomarkers capable of defining this transition could help identify patients within a therapeutically actionable window and should therefore be prioritized alongside development of CNS-directed therapies (6, 7).

Accordingly, each barrier suggests a distinct therapeutic objective. Before CNS entry, neuroinvasion is best addressed by preventing viral access to peripheral nerves through timely post-exposure prophylaxis and effective neutralization (14, 15, 74). Once neural infection is established, overcoming immune evasion requires restoration of effective antiviral immunity rather than nonspecific immune activation, potentially combining neutralizing antibodies with cellular immune mechanisms (51). After CNS dissemination, BBB restriction becomes a delivery problem, requiring brain-accessible antibodies, receptor-mediated transport, or other CNS-directed delivery strategies capable of achieving therapeutic concentrations within infected neural tissue (54, 62, 63). These approaches are therefore complementary rather than interchangeable and may need to be combined according to disease stage.

A practical translational agenda can be organized around three disease stages. In the pre-CNS stage, the goal is still to prevent neuroinvasion. Faster wound care, vaccination, risk stratification after high-risk exposures, and passive immunity remain the only consistently reliable ways to prevent symptomatic disease. Monoclonal antibodies can improve that early window. In the SYN023 phase 2b trial, 448 WHO category III exposure patients received SYN023 or human rabies immune globulin (HRIG) with vaccine; in the primary analysis group, protective rabies virus-neutralizing antibody levels were reached by Day 4 in 99.4% of SYN023 recipients versus 4.5% of HRIG recipients, and by Day 8 in 98.1% versus 12.2% (74). These are prevention data, not cure data. Once the virus has entered neural pathways, better PEP no longer solves the main problem.

In the early CNS stage, the central task changes from peripheral neutralization to CNS-accessible viral control. The immune barrier asks for a different kind of intervention: not crude inflammation, but antiviral immunity restored in the right compartment and at the right time. The F11 study is useful here because it shows that antibody therapy can do more than neutralize virus in the periphery. In Australian bat lyssavirus-infected mice, a single peripheral F11 dose at day 5 or day 7 post-infection produced 100% and 83% survival, respectively; in CVS-11 RABV infection, clinical disease and weight loss resolved in 67% of day 5-treated and 50% of day 7-treated mice (51). This points toward immune-assisted viral control, in which antibodies, Fc-dependent effector functions, and cellular immunity work together rather than in isolation.

The established encephalopathy stage is the hardest. At this point, neuroprotective and autonomic support may keep the patient alive, but support alone is not rescue. If treatment cannot reach infected CNS tissue, it is not really treatment. General BBB-delivery platforms, including receptor-mediated transcytosis and transferrin-receptor targeting, are relevant to rabies because they address delivery rather than only potency (54, 62, 75). The SynB1-conjugated antibody-cocktail study makes this concrete. A triple-antibody cocktail targeting different RABV-G epitopes, when conjugated to SynB1, achieved 80% survival in lethal CVS or dog-originated RABV models when administered at 5 dpi, after CNS invasion and symptoms were evident; the unconjugated cocktail achieved only 20% survival in CVS infection and 0% in DRV infection (52). This remains animal proof of concept, not a human standard of care. Still, it changes the question in the right direction.

Future work also needs better models and better endpoints. The One Medicine framework is attractive because naturally infected animals, especially dogs, may better approximate the timing, heterogeneity, and clinical complexity of human rabies than highly controlled rodent experiments (73). A serious preclinical platform should measure CNS exposure, viral RNA or antigen in defined brain regions, immune-cell access, neuronal function, dysautonomia, and neurological recovery (14, 15, 76). Otherwise, the field may keep producing interventions that look promising too early and fail when the biology has already moved on. Human induced pluripotent stem cell-derived neural systems, brain organoids, and, where feasible, ex vivo human neural tissue may help determine which neuronal host factors genuinely restrict RABV infection and which mechanisms observed in animal models remain relevant to human disease (**Table 1**).

**Table 1:** Cellular factors influencing RABV infection and antiviral control at the neuronal level.

Cellular factor/pathway	Main role in RABV infection	Current evidence	Key limitation/future model
RIG-I signaling	Detects viral RNA and initiates antiviral responses	Pathogenic and vaccine strains induce different RIG-I/ISG responses (37, 40, 41).	Validate in human neurons and brain organoids
IFN–STAT signaling	Restricts viral replication and promotes antiviral gene expression	RABV P protein inhibits STAT1/STAT2/STAT3 signaling (11, 12, 38, 39).	Define stage- and neuron-specific effects in human models
NF-κB signaling	Coordinates innate antiviral and inflammatory responses	RABV M protein modulates RelAp43-dependent NF-κB signaling (42, 43).	Distinguish antiviral protection from inflammatory injury
Cytoskeletal dynamics	Regulates viral internalization and intracellular trafficking	Actin remodeling and microtubule transport influence neuronal entry and spread (27, 28, 30, 31, 33)	Test whether neuronal cytoskeletal states determine susceptibility
Antigen-presenting cell/T-cell responses	Support clearance of CNS-resident virus	Dendritic-cell activation is limited; CD4^+^ T cells contribute to antibody-mediated CNS clearance (44, 50, 51).	Human CNS immune-cell co-culture and organoid models
CXCL10-associated immune recruitment	Promotes immune-cell entry into infected CNS tissue	Neuronal CXCL10 contributes to inflammatory-cell infiltration and BBB permeability (58).	Define the balance between viral clearance and neural injury
Neuronal structural integrity	Determines functional consequences of infection	Dendritic, actin, synaptic, and electrophysiological abnormalities occur without uniform neuronal loss (68–70).	Human brain organoids and ex vivo neural tissue

Symptomatic rabies is not nearly always fatal because one therapeutic ingredient is missing. It is fatal because neuroinvasion changes the battlefield, immune evasion delays the counterattack, and BBB restriction prevents rescue from arriving in time. To make symptomatic rabies treatable, rabies research must stop treating only the aftermath. It must learn how to break the barriers.

## 8 Conclusions

Symptomatic rabies is difficult to treat not because of a single therapeutic deficiency, but because neuroinvasion, immune evasion, and blood–brain barrier restriction act sequentially and cooperatively to limit effective viral clearance. This review differs from previous rabies reviews by integrating these mechanisms into a unified three-barrier framework rather than discussing neuropathogenesis, immune escape, and CNS drug delivery as separate problems. The framework also links each biological barrier to a corresponding therapeutic window, emphasizing that successful post-symptom treatment will likely require stage-specific combinations of CNS-accessible antiviral therapy, controlled immune engagement, and neuroprotective support. Although most mechanistic and therapeutic evidence remains derived from experimental models, organizing symptomatic rabies around these interacting barriers may help identify where current interventions fail and where future translational studies should focus.

Several limitations should be acknowledged. Much of the mechanistic and therapeutic evidence reviewed here is derived from cellular and animal models, whereas direct validation in symptomatic human rabies remains very limited. Important differences in disease timing, viral strain, CNS distribution, immune responses, and therapeutic delivery may limit the direct translation of experimental findings to patients. Future research should therefore prioritize early identification of CNS involvement, human-relevant neural and organoid models, and stage-specific evaluation of CNS-accessible antiviral and immune-based therapies. Clinically, these findings emphasize the importance of rapid diagnosis and referral, while experimental rescue strategies should be evaluated within appropriately controlled translational and clinical research frameworks.
